# Evaluation of a ^99m^Tc-tricine Vascular Disrupting Agent as an *In-vivo* Imaging in 4T1 Mouse Breast Tumor Model 

**Published:** 2017

**Authors:** Mostafa Erfani, Seyed Pezhman Shirmardi, Mohammad Shafiei

**Affiliations:** a *Radiation Application Research School, Nuclear Science and Technology Research Institute (NSTRI), Tehran, Iran. *

**Keywords:** Deacetylcolchicine, Labeling, Pharmacokinetics, Tricine, 4T1 Tumor

## Abstract

Colchicine as a vascular disrupting agent creates microtubule destabilization which induces vessel blockage and consequently cell death. Accordingly, colchicines and its analogues radiolabeled with ^99m^Tc may have potential for visualization of tumor. In this work, deacetylcolchicine a colchicine analogue was labeled with ^99m^Tc via tricine as a coligand and characterized for its tumor targeting properties. The *in-vitro* radiochemical stability and the biodistribution were studied in 4T1 breast tumor model bearing mice. Labeling yield of more than 90% was obtained corresponding to a specific activity of 46 MBq/µmol. *In-vivo* biodistribution studies demonstrated that radiocomplex had high tumor to muscle and tumor to blood ratios at early time points. Planer gamma imaging of tumor bearing mice showed that this radioconjugate was able to clearly visualize tumors. According to high tumor uptake, presented radiocomplex may have a potential for targeted imaging studies.

## Introduction

In spite of the remarkable advances in research and treatment, cancer is one of the main health problems with high mortality and morbidity globally. Cancer is currently ranked among the second leading cause of death worldwide, being only exceeded by cardiovascular diseases (1). Early detection of cancer enables treatment of primary tumor, while metastatic tumor is very difficult to manage, resulting in dramatically reduced life expectancy.

Colchicum autumnale plant extract containing colchicine has been used to treat gout for more than 2000 years. The maximum oral dose in gout attacks is 4 mg over the first 24 h, and the dosage should be decreased stepwise over the next 2 days. Colchicine is effective in preventing the recurrent attacks of fever that characterize familial Mediterranean fever (FMF) or periodic disease. Oral colchicine in a daily dosage of 0.5-2 mg decreases both the intensity and the frequency of the attacks and missing a dose often starts an attack. Colchicine has been suggested in the treatment of other inflammatory diseases such as Behcet disease, several vasculitides, and pericarditis. Colchicine is also used in primary biliary cirrhosis and other forms of cirrhosis, immune thrombocytopenic purpura and a number of skin diseases characterized by neutrophil infiltration (2).

 The chief mechanism of action of colchicine is inhibition of microtubule polymerization. Each colchicine molecule binds to a tubulin molecule, preventing it from incorporating into the polymer. Microtubule elongation stops, the mitotic spindle is disrupted, and cell division cannot proceed. This effect makes colchicines one of the oldest identified antimitotic drugs (3). 

Conjugation of colchicine to imaging agents offers the facility to monitor its physiological distribution and effectiveness as vascular disrupting agent. The ^99m^Tc-labelled ethylenedicysteine conjugate with colchicine is illustrated of efforts which have been made to achieve such conjugate structures in nuclear medicine. Numerous colchicine analogs have been radiolabeled for tumor imaging (4-10), however, development of a facile and stable method for labeling with ^99m^Tc remains as an active area of research.

Techentetium-99m (^99m^Tc) is extensively used for diagnosis in nuclear medicine, mainly due to its physical properties and availability, including a 6 h half-life, a gamma decay emission of 140 keV, ready availability, and low cost. The favorable physical and imaging characteristics of this radionuclide have stimulated the interest in labeling different molecules with ^99m^Tc for the development of targeted molecular imaging agents. One bifunctional chelator (BFC) that has gained great attention is the 6-hydrazinonicotinyl group known as HYNIC, first described by Abrams *et al.* (11). HYNIC can coordinate to ^99m^Tc through its pyridyl nitrogen and hydrazine nitrogen (12), with the additional requirement of a co-ligand to complete the coordination sphere around the metal (13, 14). HYNIC can be coupled via an activated carboxylic function to free primary amine groups of the molecule (15-18).

Colchicine and its metabolites are eliminated through the bile and kidneys and the biliary excretion may be the main route of colchicines elimination. P-glycoprotein, which is strongly expressed by bile-ductule membranes, plays a key role in this excretion (19, 20). Due to biliary excretion and gastrointestinal clearance, limitation of detection for tumors or metastasis in abdominal region due to low tumor to background activity exists. In this work, deacetylcolchicine as a colchicine analogue has been labeled with ^99m^Tc via tricine coligand. The optimum radiolabeling conditions and further characterization of the ^99m^Tc-tricine-HYNIC-Deacetylcolchicine (^99m^Tc-tricine-ligand) using an *in-vivo* C57 mice bearing 4T1 mouse breast tumor model have been presented. 

## Experimental

*General*

All chemicals were purchased from Sigma-Aldrich Co. and used without further purification. The 4T1 mammary tumor cell was purchased from Pasteur Institute (Tehran, Iran). The cell culture medium was Roswell Park Memorial Institute (RPMI-1640) supplemented with 10% fetal bovine serum (FBS), amino acids, vitamins and penicillin/streptomycin (Gibco, Eggenstein, Germany). Sodium pertechnetate (Na^99m^TcO_4_) obtained from commercial ^99^Mo/^99m^Tc generator (Radioisotope Division, AEOI). High performance liquid chromatography was performed on a JASCO 880-PU intelligent pump HPLC system equipped with a multiwavelength detector and a flow-through Raytest-Gabi gamma-detector. CC 250/4.6 Nucleosil 120-5 C18 column was used for analytical HPLC. The gradient systems consisted of 0.1% trifluoroacetic acid/water (Solvent A) and acetonitrile (Solvent B). A Gradient program was used: 0 min 95% A (5% B), 5 min 95% A (5% B), 25 min 0% A (100% B), 30 min 0% A (100% B), 35 min 95% A (5% B), flow = 0.75 mL/min, λ = 280 nm. Mass spectrum was recorded on an Agilent 1100/ Bruker Daltonic (Ion trap) VL instrument. ^1^H NMR spectrum obtained on Bruker 500 MHz NMR spectrometer Quantitative gamma counting was performed on an EG&G / ORTEC Model 4001M Mini Bin & Power Supply counter. 

*Chemical Synthesis*

Deacetylcolchicine was synthesized according to the previously reported method by Lagnoux *et al.* (21). Hydrazinopyridine-3-carboxylic acid (HYNIC) in the form of Boc-HYNIC (2 mmol), and deacetylcolchicine (1 mmol) were added to a round bottom flask, and to this, dry dimethylformamide (2 mL) followed by 2 mmol of 2-(7-aza-1H-benzotriazole-1-yl)-1,1,3,3-tetramethyluronium hexafluorophosphate (HATU) and 6 mmol of N, N-diisopropylethylamine (DIPEA) were added and stirred at 25 °C for 6 h. Water (20 mL) and ethylacetate (20 mL) were added to the reaction mixture and organic fraction removed and evaporated in vacuum. Afterwards, to leaving yellow solid was added mixture of CH_2_Cl_2_ (4 mL)/ TFA (1 mL) incubated at 25 °C for 2 h. The mixture was evaporated in vacuum and was added to the residue diisopropyl ether (10 mL) to yield an off-white solid precipitate HYNIC-Deacetylcolchicine. ^1^H NMR (500 MHz, CDCl_3_) δ : 8.74 (*d*, *J* = 2.4 1H), 7.91 (*dd*, *J* = 2.4, 8.8, 1H), 7.71 (*s*, 1H), 7.47 (*d*, *J* = 11, 1H), 7.03 (*d*, *J* = 11, 1H), 6.79 (*d*, *J* = 8.8, 1H), 6.57 (*s*, 1H), 4.12 (*m*, 1H), 3.92 (*s*, 3H), 3.82 (*s*, 3H), 3.81 (*s*, 3H), 3.53 (*s*, 3H), 2.58 (*m*, 2H), 2.40 (*m*, 2H). MS (ESI+) calculated for C_26_H_28_N_4_O_6 _: *m/z* 492.20; found, 492.5 (M+H)^+^. 

**Table 1:** Analytical data of HYNIC-deacetylcolchicine. Analytical HPLC performed on a C18 column using a gradient of 0.1% aqueous TFA (Solvent A) in acetonitrile (Solvent B) in 30 min at 1 mL/min. The following gradient was used. A:B ratio of: 95:5 at 0 min, 95:5 at 5 min, 0:100 at 25 min, 0:100 at 27 min, 95:5 at 30 min

**Compound**	**Mass spectrum**		**RP-HPLC**	
	**Calculated mass (g/mol)**	**Observed mass (g/mol)**	**Retention Time (min)**	**Purity** **(%)**
HYNIC-ligand	492.20	492.5 [M+H]^+^	19.26	> 98.5

**Table 2:** Biodistribution studies of ^99m^Tc-tricine-ligand in mice (% injected dose per gram organ ± SD, n = 3).

**24 (h)**	**4 (h)**	**1 (h)**	**Organ**
0.04 ± 0.12	1.35 ± 0.37	3.23 ± 0.80	Blood
0.21 ± 0.14	3.85 ± 0.33	5.35 ± 0.41	Kidneys
0.11 ± 0.03	0.57 ± 0.06	1.23 ± 0.15	Spleen
0.07 ± 0.01	0.56 ± 0.10	0.97 ± 0.14	Stomach
0.11 ± 0.02	1.56 ± 0.34	11.25 ± 1.03	intestines
0.20 ± 0.05	1.35 ± 0.18	4.13 ± 0.39	Liver
0.1 ± 0.03	0.61 ± 0.12	3.21 ± 0.22	Lung
0.05 ± 0.01	0.45 ± 0.04	1.62 ± 0.21	Muscle
0.15 ± 0.06	1.05 ± 0.17	3.59 ± 0.23	Tumor
2.6	2.3	2.2	Tumor/Muscle
3.75	3.0	1.1	Tumor/Blood

**Figure 1:**
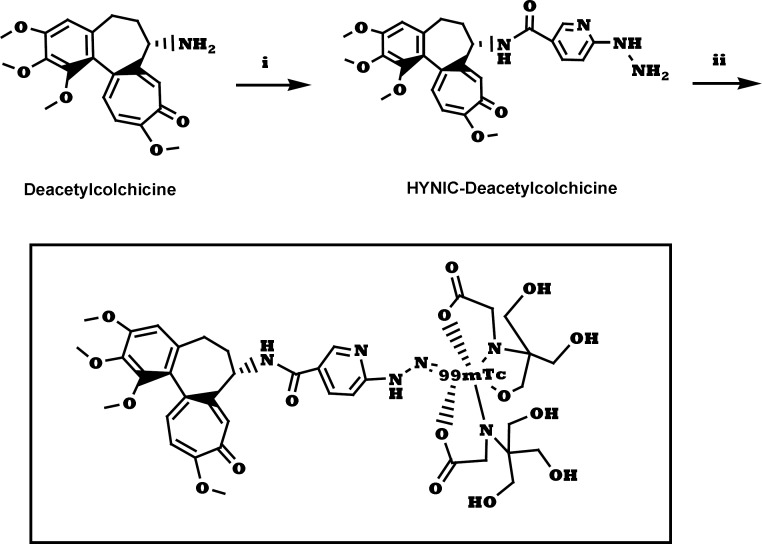
Synthesis of ^99m^Tc-tricine-ligand. (i) a. HYNIC-Boc, HATU, DIPEA, DMF, 25 °C, 6 h. b. TFA, CH_2_Cl_2_, 25 °C, 2 h; (ii) ^99m^TcO_4_^-^, tricine, 90 °C, 10 min.

**Figure 2:**
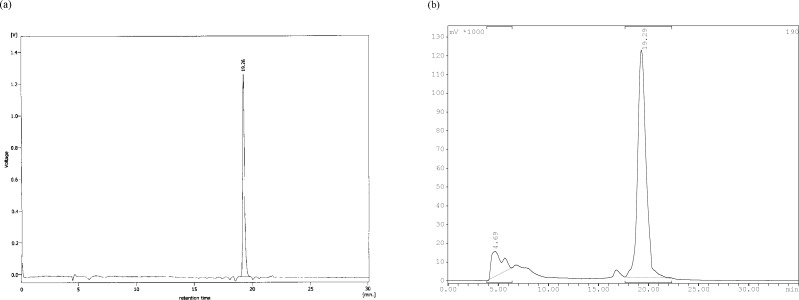
Reverse phase HPLC of radiolabeled ligand. (a) ^99m^Tc-tricine-ligand in multiwavelength detector (λ = 280 nm) and (b) for radiocomplex in Raytest-Gabi gamma-detector. CC 250/4.6 Nucleosil 120-5 C-18 column from Teknokroma was used. 0.1% trifluoroacetic acid/water (Solvent A) and 0.1% trifluoroacetic acid/acetonitrile (Solvent B) were used as a mobile phase in the following gradient: 0 min 95% A (5% B), 5 min 95% A (5% B), 25 min 0% A (100% B), 30 min 0% A (100% B), flow = 1 mL/min

**Figure 3:**
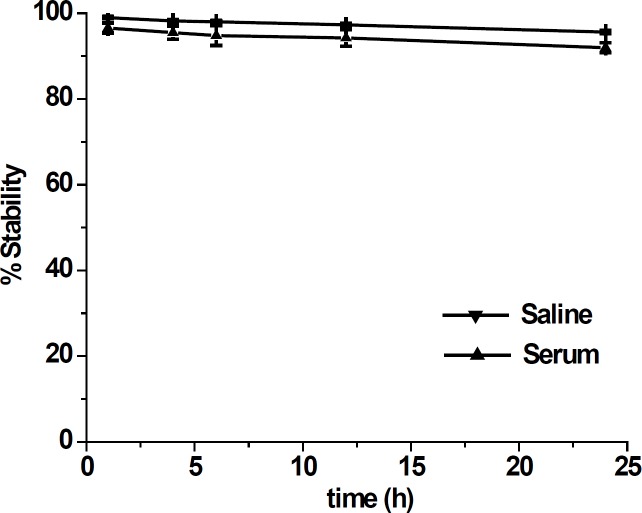
*In-vitro* stability of ^99m^Tc-tricine-ligand in saline solution and serum. The data are expressed as mean ± standard deviation (n = 3

**Figure 4:**
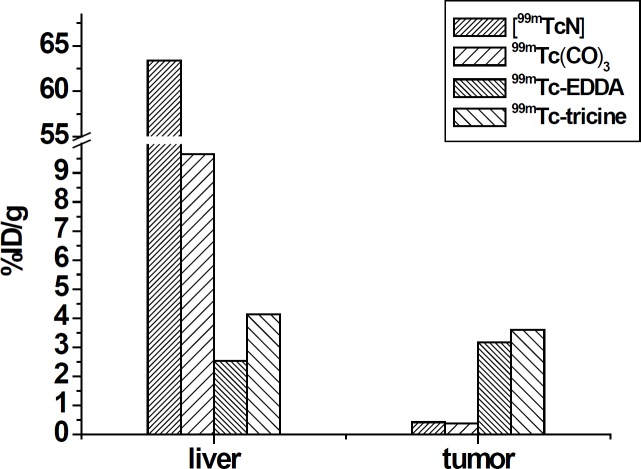
The liver and tumor uptake values for colchicine analogs when labeled through different labeling methods

**Figure 5:**
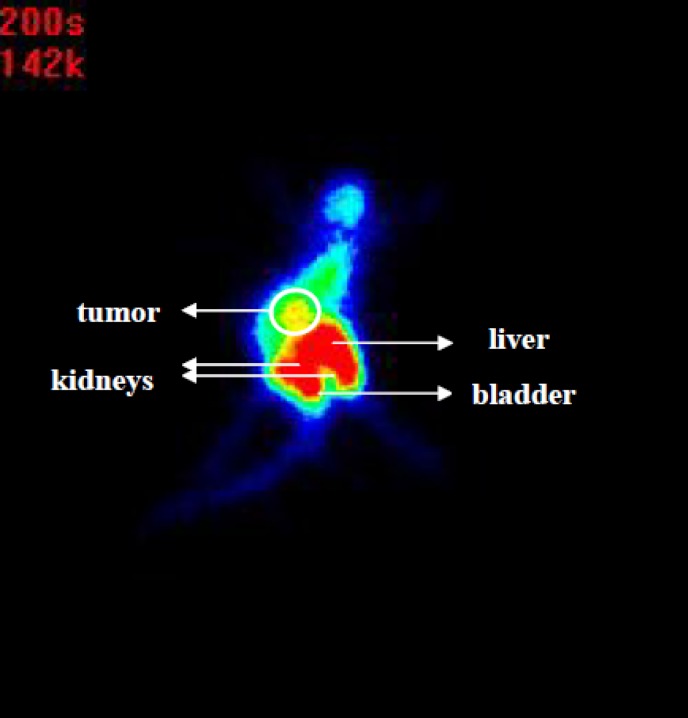
Posterior image ^99m^Tc-tricine-ligand in C57 mice bearing 4T1 breast tumor at 1 h post injection

^99m^ *Tc labeling via tricine*

A stock solution of HYNIC-Deacetylcolchicine (1 mg/mL) was prepared by dissolving in distilled water. In order to optimize labeling efficiency a series of studies as the following were performed: changing the amount of ligand, varying the amount of tricine as a coligand, altering the amount of SnCl_2 _as a reducing agent, and adjustment of the reaction pH. In addition, the effect of various amounts of ^99m^TcO_4_^-^ used for the labeling reaction was investigated.

Radiolabeling was performed by adding 1-20 µL of the stock solution of ligand and 5-40 mg of tricine to 0.5 mL of water in a vial. To this solution 10-100 µg SnCl_2 _was added. Finally, 185-1110 MBq of ^99m^TcO_4_^-^ in 0.5 mL saline was added to the solution and the pH was adjusted. The vial was incubated at 90 °C for 10 min and then cooled down to room temperature. 

*Labeling analysis and stability*

^99m^Tc-tricine-ligand was characterized by analytical RP-HPLC and TLC on silica gel 60 (Merck) using different mobile phases: 2-butanone for free ^99m^TcO_4_^-^ (R_f_ = 1), and water/acetonitrile 1/1 for ^99m^Tc colloid (R_f_ = 0). The radioactivity was quantified by cutting the strip (1.5 × 10 cm^2^) into 1 cm pieces and counting in a well type gamma counter. 

The stability of ^99m^Tc-tricine-ligand in saline solution was evaluated by incubation of the reaction mixture at room temperature (25 °C) up to 24 h. Stability in human serum at 37 °C was tested in parallel after adding 100 μL of reaction mixture to 1 mL of fresh human serum. The incubation mixtures were sampled at 1, 4, 6, 12 and 24 h time points. Serum samples (100 - 200 μL) were treated with ethanol (200 - 400 μL) and centrifuged (4000×g, 5 min, 4 °C) to precipitate the serum proteins. 20-100 μL aliquots from the supernatant were separated to assess the degradation of ^99m^Tc-tricine-ligand by HPLC. 

*Log P values*

In a 2 mL micro tube, 0.5 mL of the ^99m^Tc-tricine-ligand in PBS was mixed with 0.5 mL of octanol. The tube was vigorously vortexed over a period of 10 min and centrifuged at 4000×g for 5 min. Three aliquots of 100 μL were sampled from each layer and counted in the Gamma-counter. The averaged activities from the aqueous and the octanol layers were used to calculate the log P values. Finally, by dividing the counts of the octanol phase by that of the aqueous phase octanol-to-water partition coefficient (P_o/w_) of the radioligand was calculated. 

*Biodistribution*

Animal experiments were performed in compliance with the regulations of our institution and with generally accepted guidelines governing such work. The 4T1 mammary tumor cell was cultured in RPMI 1640 supplemented with 10% (v/v) FBS, glutamine (2 mM), penicillin (50 U/mL) and streptomycin (50 μg/mL). Cells were maintained in a humidified 5% CO_2_/air atmosphere at 37 °C. A suspension of 4T1 cells (1×10^7^) in PBS (0.1 mL) was subcutaneously injected in the right flank of each mice. Seven to ten days after inoculation the tumors developed. Totally 9 mice bearing 4T1 breast tumor into three groups (each group three mice) received 37 MBq of radioligand in 100 µL of saline via a tail vein. After 1, 4 and 24 h, the mice were killed, organs of interest were collected, weighed and radioactivity was measured in a gamma-counter. The percentage of the injected dose per gram (% ID/g) was calculated for each tissue. 

*Scintigraphy studies*

In order to better understand the whole body localization, the behavior of ^99m^Tc-tricine-ligand, was evaluated by the static images of the mice, each of which received (20 MBq, 100 µL) of radioligand via a tail vein. Before that the imaging mice was anesthetized with 0.05 mL ketamine 10% (3.3 mg) and 0.05 mL xylazine 2% (1.33 mg) intraperitoneally. After about 5 min, the animal was fixed on a board by being covered with pieces of cloth for immobilization during the scanning. Scintigraphy imaging study was obtained using a single head gamma camera (small area mobile, 140 keV, Siemens, Germany) equipped with high sensitivity parallel whole collimator. Whole body image has been obtained using a 256 × 256 matrix size with 5000 kilo counts at 1 h post injection. For image acquisition, a 10% acceptance window around the 140 keV photo peak was used. 

## Results and Discussion

HYNIC-Deacetylcolchicine was obtained in an overall yield of 30% and was characterized by 1H, MS spectroscopy, and analytical RP-HPLC in above mentioned analytical condition (Table 1). The prepared ligand is excellent candidate due to the achievement of labeling in high specific activity followed by using various coligands.

To develop a radioligand, an optimal combination of individual ingredients including ligand, SnCl_2_, and tricine was systematically examined. The amount of ligand used in the labeling is important, because the excess of free ligand may lead to an increased risk of inducing pharmacologically undesired side effects. Colchicine is effective in a dose of 0.015 mg/kg, toxic in doses greater than 0.1 mg/kg, and lethal in a dose of 0.8 mg/kg (2). Amount of ligand up to 10 µg was used for the labeling to insure presence of sufficient amount of ligand for complex formation and also to insure that colchicine was used in safe dose. The optimal amounts of reducing agent SnCl_2_ and tricine as a coligand were found to be 20-60 µg and 10-30 mg respectively.

The labeling performed by using 10 µg ligand, 20 mg tricine, 40 µg stannous chloride dehydrate, and 925 MBq sodium pertechnetate as a labeling agents at a pH = 5, and a high labeling yield and a specific activity of 46 MBq/nmol for ^99m^Tc-tricine-ligand were achieved. The suggested structure for desired complex is shown in Figure 1.

In the radiochemical purity determination of radiocomplex by ITLC, in the first part a solvent system which consisted of acetone, main part of activity remained in the origin which related to radiocomplex and less than 5% of total activity was moved and counted in R_f_ = 1 which belonged to ^99m^TcO_4_^-^. In the second part choosing water/acetonitrile 1/1 only minimal activity (less than 3%) remained in the origin corresponding to reduced technetium-99m and main part of activity was moved and counted in R_f_ = 1 which related to radiocomplex. The radiocomplex was characterized by HPLC which prepared in >90% yield. Two different peaks with retention times of 4.69 min and 19.29 min were observed, as shown in Figure 2. The ^99m^Tc-tricine-ligand has been characterized by comparison with the corresponding ligand. The UV-HPLC chromatogram retention time of ligand (Figure 2a) was observed to be 19.26 min, which matches well with 19.29 min gamma-HPLC chromatogram retention time of radiocomplex (Figure 2b). The peak at 4.69 min is assigned to ^99m^TcO_4_^-^ and the peak at 19.29 min the ^99m^Tc-tricine-ligand. 

Stability studies in saline solution showed a good stability of ^99m^Tc-tricine-ligand (> 90%) with no considerable release of ^99m^TcO_4_^-^ or ligand degradation during the observation time (Figure 3). In serum stability studies, the ethanol fraction was characterized by HPLC where a single peak was observed at the same time as that of the complex, indicating no decomposition of the ^99m^Tc-tricine-ligand. Achievement of a high labeling yield without any further indication of impurities is in accordance with previously reported studies regarding usage of tricine as a co-ligand (13, 14). 

The calculated partition coefficient (log P) for ^99m^Tc-tricine-ligand was -1.23 ± 0.18. This result shows that radiocomplex is slightly hydrophilic due to the presence of polar groups which are present in the complex structure. The ^99m^Tc coligand also involved in the hydrophilicity plays an important role in *in-vitro* behavior of ^99m^Tc-tricine-ligand. 

A biodistribution characteristic of ^99m^Tc-tricine-ligand was evaluated using mice bearing 4T1 breast tumor xenografts. The data are summarized in Table 2. The complex was excreted via hepatobiliary and renal pathway. Uptake values at 1 h in intestines (11.25 ± 1.03% ID/g) were higher than liver uptake (4.13 ± 0.39% ID/g) which confirms the fast hepatic transit. Recently it has been established that P-glycoprotein expressed by the biliary ductules in the liver can excrete colchicine and biliary excretion may be the main route of colchicines elimination (19, 20). Moreover, P-glycoprotein expression by the proximal renal tubule increases the amount of colchicines excreted in urine. 

In connection to blood uptake the activity of 3.23 ± 0.80% ID/g at 1 h could be ascribed to percentage of its bound radiocomplex to plasma proteins which could be related to conjugate lipophilicity and also tricine coligand.^ 99m^Tc-tricine-ligand also showed high uptake values in the tumor (3.59 ± 0.23% ID/g) at 1 h after injection with increasing in tumor/blood ratio during the observed period. 

Labeling of colchicines derivatives using ^99m^Tc carbonyl and ^99m^Tc nitride cores was reported by Korde *et al*. (7). They reported significant liver accumulation (63.4% ID/organ at 1 h) with slow hepatic clearance and not very significant tumor uptake (0.42% ID/g at 1 h) For [^99m^TcN]-colchicine complex. Wang *et al*. also reported ^99m^Tc(CO)_3_-labeling of a novel colchicines complex via click reaction (10). They reported still considerable liver (9.64% ID/g at 1 h) uptake and low tumor uptake (0.37% ID/g at 1 h). They concluded that further modification on the linker and ^99m^Tc-chelate will be necessary to improve targeting efficacy and pharmacokinetic profile of colchicines. Colchicine has previously been labeled by ^99m^Tc via EDDA coligand and through exchange labeling method showing 2.53% ID/g uptake in liver and the tumor uptake that was 3.17 ± 0.14% ID/g at 1 h post 

injection (9). 

In comparison to EDDA coligand, for ^99m^Tc-tricine-ligand the activity uptake in liver was increased (2.53% ID/g versus 4.13% ID/g) and the tumor uptake increased (3.17% ID/g versus 3.59% ID/g) at 1 h post injection respectively.

It seems that the pharmacokinetic profile of ^99m^Tc-tricine-ligand compared to ^99m^Tc carbonyl and ^99m^Tc nitride labeling colchicine derivatives with respect to uptake reduction for the liver and gastrointestinal was improved, while this value was not improved compared to EDDA. Compared to all of the above mentioned previously reported labeling methods, the tumor uptake value of the ^99m^Tc-tricine-ligand increased which lead to an increase in the tumor/blood ratio (Figure 4). 

Tubulin is the main molecule in tumor cells which plays a key role in cell transport and division (21). Colchicine prevents tubulin polymerization and thereby disrupts microtubule function which can be evaluated by single photo imaging after use of labeled colchicine. Through scintigraphy it was observed that the ^99m^Tc-tricine-ligand mainly was accumulated in the tumor, liver, and kidneys (Figure 5). Scintigraphy images also support the results obtained by biodistribution studies. According to high tumor uptake and also high uptake in liver and intestine which could be contributed to the fast hepatic transit of radiocomplex, scintigraphy at the earlier time points (up to 1 h) may resolve limitation of detection for tumors or metastasis in abdominal region.

## Conclusion

^99m^Tc-tricine-vascular disrupting agent prepared with high labeling yield of more than 90% and high stability and no significant impurities were detected. Accumulation in tumor followed by excretion via the kidney and hepatic pathway were observed. The tumor specificity of ^99m^Tc-tricine-ligand provides further understanding toward its possible usefulness for imaging tumor states. 
